# Deep extreme learning machine with knowledge augmentation for EEG seizure signal recognition

**DOI:** 10.3389/fninf.2023.1205529

**Published:** 2023-08-24

**Authors:** Xiongtao Zhang, Shuai Dong, Qing Shen, Jie Zhou, Jingjing Min

**Affiliations:** ^1^School of Information Engineering, Huzhou University, Huzhou, China; ^2^Zhejiang Province Key Laboratory of Smart Management and Application of Modern Agricultural Resources, Huzhou University, Huzhou, China; ^3^Department of Computer Science and Engineering, Shaoxing University, Shaoxing, China; ^4^Department of Neurology, The First People's Hospital of Huzhou, First Affiliated Hospital of Huzhou University, Huzhou, China

**Keywords:** multilayer extreme learning machine, deep network, knowledge utilization, EEG, seizure recognition

## Abstract

**Introduction:**

Intelligent recognition of electroencephalogram (EEG) signals can remarkably improve the accuracy of epileptic seizure prediction, which is essential for epileptic diagnosis. Extreme learning machine (ELM) has been applied to EEG signals recognition, however, the artifacts and noises in EEG signals have a serious effect on recognition efficiency. Deep learning is capable of noise resistance, contributing to removing the noise in raw EEG signals. But traditional deep networks suffer from time-consuming training and slow convergence.

**Methods:**

Therefore, a novel deep learning based ELM (denoted as DELM) motivated by stacking generalization principle is proposed in this paper. Deep extreme learning machine (DELM) is a hierarchical network composed of several independent ELM modules. Augmented EEG knowledge is taken as complementary component, which will then be mapped into next module. This learning process is so simple and fast, meanwhile, it can excavate the implicit knowledge in raw data to a greater extent. Additionally, the proposed method is operated in a single-direction manner, so there is no need to perform parameters fine-tuning, which saves the expense of time.

**Results:**

Extensive experiments are conducted on the public Bonn EEG dataset. The experimental results demonstrate that compared with the commonly-used seizure prediction methods, the proposed DELM wins the best average accuracies in 13 out of the 22 data and the best average F-measure scores in 10 out of the 22 data. And the running time of DELM is more than two times quickly than deep learning methods.

**Discussion:**

Therefore, DELM is superior to traditional and some state-of-the-art machine learning methods. The proposed architecture demonstrates its feasibility and superiority in epileptic EEG signal recognition. The proposed less computationally intensive deep classifier enables faster seizure onset detection, which is showing great potential on the application of real-time EEG signal classification.

## 1. Introduction

Epilepsy is a common chronic neurological disease caused by sudden abnormal discharge of neurons in human brain (Sanei and Chambers, 2013). Most epileptic patients have no difference from common people when epileptic seizure does not appear, but epilepsy has a serious effect on quality of human life, or even causes fatal harm (Iasemidis et al., 2003). Rapid and accurate diagnosis of epilepsy is essential for the treatment of patients and the risk reduction of potential seizures, and its relevant technique is urgently expected in current society. Electroencephalogram (EEG) shows the electrical activity of human brain recorded by amplifying voltage differences between electrodes placed on the scalp or cerebral cortex. In traditional epilepsy detection by doctors, visual marking of long EEG recordings is a tedious and high-cost task with high misjudgment rate, especially taking into account the subjectiveness of experts (Wang et al., 2018).

EEG signal recognition plays an important role in the assessment and auxiliary diagnosis of epilepsy (Ghosh-Dastidar et al., 2007; Ahmadlou and Adeli, 2011; Ayman et al., 2023). Careful analysis of the electroencephalograph records can provide valuable insight and improved understanding of the mechanisms causing epileptic disorders. Machine learning methods, such as neural network (Subasi and Ercelebi, 2005; Kumar et al., 2010), fuzzy system (Güler and Übeyli, 2005), support vector machine (Panda et al., 2010; Nicolaou and Georgiou, 2012; Kumar et al., 2014), and extreme learning machine (Liang et al., 2006b; Yuan et al., 2011; Song and Zhang, 2013), have been extensively used in EEG signal recognition. But some of the existing intelligent methods perform poor in terms of classification accuracy, real-time prediction and so on. As a novel paradigm of learning method, ELM can not only learn rapidly with good generalization performance, but also effectively overcome the inherent drawbacks of some intelligent technologies. In recent years, ELM and its variants (Huang et al., 2004, 2006, 2011a,b; Liang et al., 2006a; Betthauser et al., 2017) have received increasing attention. However, its shallow structure is deficient in extracting the significant implicit information from the original data, which becomes the main bottleneck restricting its development. As a popular trend in machine learning, deep learning has confirmed that pattern recognition can remarkably benefit from the knowledge learned via hierarchical feature representation. Typical deep networks include deep belief network (Hinton and Salakhutdinov, 2006; Hinton et al., 2006; Plis et al., 2014), convolutional neural network (Khan et al., 2017; Acharya et al., 2018; Choi et al., 2019), stack autoencoder (Bengio et al., 2007; Vincent et al., 2010; Xu et al., 2015), etc. There are many artifacts and noises in EEG signals, which can seriously decrease recognition efficiency (Bengio, 2009; Zhou and Chan, 2016; Bhattacharyya and Pachori, 2017). Deep learning is exactly able to resist noise in recognition process and can remove noise from EEG data (Huang et al., 2013; Deng et al., 2016). However, conventional deep learning algorithm is time-consuming with complicated structure and can easily lead to overfitting in presence of limited available samples. In order to tackle the aforementioned problems, ELM is gradually combined with deep learning to generate a high-performance model (Tang et al., 2014, 2015; Yu et al., 2015; Zhu et al., 2015; Duan et al., 2016; McIntosh et al., 2020). However, most of the existing hierarchical ELM models can hardly effectively use the knowledge learned in previous layers.

ELM is popular for its high-speed response, real-time prediction ability, network conciseness, and excellent generalization performance. The thought of deep learning can be beneficial to excavate the invisible value of input to the greatest extent. To address the problem of lacking representational learning, deep extreme learning machine (DELM) is proposed to recognize EEG epileptic signals. The efficient deep classifier is based on stacked structure, which in essence consists of several modules whose hidden layer parameters are initialized randomly. The proposed method forms a hierarchical structure to aggregate some discrete and valuable information stepwisely into knowledge for hierarchical representation. The previous valuable information is fed into new input in the manner of available knowledge and then transmitted to current sub-model, which serves to implement the subsequent recognition task better. According to stacking generalization theory, the output of the next sub-model plus the knowledge of the previous sub-model in DELM can indeed open the manifold structure of the input space, which resulting an improved performance. DELM have accomplish fast epileptic recognition and show greater performance in EEG signal classification than traditional ELM and some of the state-of-the-art methods, which makes it possible to finish accurate epilepsy diagnosis in real time and with high precision. The main contributions of this work are as follows:

(1) DELM is a novel deep learning structure, which is the product of the fusion of ELM and deep learning. DELM is composed of original ELMs, accordingly, the new structure is inherently brief, flexible to implement, and demonstrates a superior learning performance. Additionally, the introduction of deep representation ensures that valuable knowledge is refined and not wasted. Learning rich representations efficiently is crucial for achieving better generalization performance and informative features can promote the accuracy. In our paper, the new framework can achieve classification accuracy comparable to that of existing deep network schemes in EEG recognition tasks, while DELM takes the leading position in training speed.
(2) Motivated by deep learning, the proposed DELM is used to capture useful information in multi-dimensional EEG variables. DELM is a hierarchical framework, which incorporates a stepwise knowledge augmentation strategy into original ELM. It learns knowledge in an incremental way and expands it in the manner of forward calculation. The current sub-model can exploit knowledge from all previous sub-models and the recognition results can be obtained in the last layer.
(3) DELM uses classic ELM as the basic building block, and each module is the same as the original ELM structure. Supervised learning performs throughout the whole learning process and each sample has a tendency to approach to its own class under the supervision.

The main differences of the proposed DELM and traditional and deep learning methods are summarized in Table 1. The rest of this paper is organized as follows. Section 2 presents the details of deep extreme learning machine proposed in our work and describes its learning process. Section 3 introduces the experiment conducted and compares the recognition performance of the proposed method with that of existing conventional methods on real EEG datasets. Finally, Section 4 concludes the findings of the study.

**Table 1:** Comparisons between the proposed DELM and traditional and deep learning methods.

**Models**	**Running speed quickly**	**Deep learning ability**
ELM	Yes	No
Adaboost	No	No
DBN	No	Yes
SAE	No	Yes
DELM	Yes	Yes

## 2. The proposed classifier DELM

### 2.1. The proposed architecture based on deep representation

ELM with *L* hidden neural units and activation function g(.) can approximate these *N* samples with zero error, which is modeled as (Huang et al., 2004):


(1) $$\begin{array}{l} \overset{L}{\underset{i=1}{\sum }}\beta_{i}\text{g}\left(w_{i}x_{j}\text{+}b_{i}\right)=t_{j},j=1,...,N \end{array}$$

where $x_{i}={\left[x_{i1},x_{i2},\ldots ,x_{in}\right]}^{T}\in R^{n}$, $t_{i}={\left[t_{i1},t_{i2},\ldots ,t_{im}\right]}^{T}\in R^{m}$, *β*_*i*_ is the weight vector connecting the ith hidden node and the output nodes, *w*_*i*_ is the *i*th hidden node and the input nodes, and *b*_*i*_ represents the bias of the *i*th hidden node. For the sake of convenience, the equation can be written in a compact form


(2) $$\begin{array}{l} H\beta =T \end{array}$$

with $\beta ={\left[\beta_{1},\ldots ,\beta_{L}\right]}_{m\times L}^{T}$, $T={\left[t_{1},\cdots \;,t_{N}\right]}_{m\times N}^{T}$ and $\begin{array}{l} H(w_{1},...,w_{L},b_{1},...,b_{L},x_{1},...,x_{N})\\ ={\left(\begin{array}{ccc} \text{g}(w_{1}\cdot x_{1}\text{+}b_{1}) & \ldots & \text{g}(w_{L}\cdot x_{1}\text{+}b_{L})\\ \vdots & \cdots & \vdots \\ \text{g}(w_{1}\cdot x_{N}\text{+}b_{1}) & \cdots & \text{g}(w_{L}\cdot x_{N}\text{+}b_{L}) \end{array}\right)}_{N\times L} \end{array}$

where *H* is the hidden layer output matrix of neural network, the *i*th column of *H* is corresponding output of the *i*th hidden layer unit with respect to inputs.

The solution of Equation 2 is equivalent to the next optimization problem (Liang et al., 2006a):


(3) $$\begin{array}{l} \left\|H(w_{1},...,w_{L},b_{1},...,b_{L})\hat{\beta }-T\right\|\\ =\underset{\beta }{\min}\left\|H(w_{1},...,w_{L},b_{1},...,b_{L})\beta -T\right\| \end{array}$$

In most cases of practical application, the hidden layer neurons is far less than the samples need be trained, *L*≪*N*. The output matrix of the hidden layer is not a square matrix, and the minimum norm least-squares solution of the above linear system can be calculated by Equation 4 (Huang et al., 2011a):


(4) $$\begin{array}{l} \hat{\beta }\text{=}H^{+}T \end{array}$$

*H*^+^ denotes the Moore-Penrose generalized inverse of the output matrix *H*. The theory of ELM is aimed at reaching not only the smallest training error but also the smallest norm of output weights.

**Figure 1:**
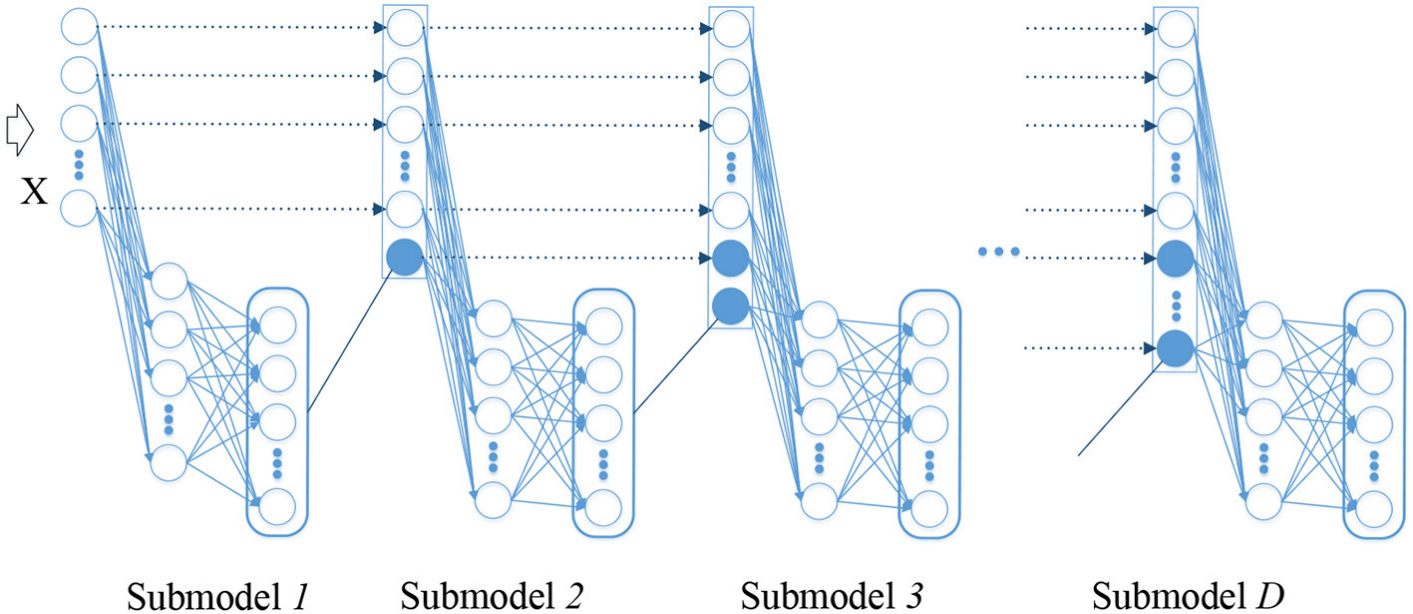
The proposed hierarchical architecture.

ELM is a shallow network composed of three layers (respectively input layer, hidden layer and output layer), whose representation capability is limited. Adequate representation of the input is routinely desired to acquire an excellent performance in the idea of deep learning. On account of the flexibility and efficiency of ELMs, ELM is extended to the learning of deep neural network (DNN) to shorten the learning time dramatically and reduce the computational complexity without deserting their original excellence. The proposed architecture constructed from ELM building block is a new ELM-based stacked structure that processes information layer by layer in order to utilize the learned knowledge. Figure 1 depicts the architecture of the proposed hierarchical method.

The proposed structure inherits the simplicity of the original ELM, and then digestion and absorption of knowledge is performed in multiple sub-model. In DELM, the initial EEG epileptic signal is learned step by step in a forward manner. The representation learned from the previous layer is regarded as new knowledge and will then be taught. Upon the arrival of given input, the corresponding linear system can be solved immediately in the first ELM.

In a singleton ELM module, the knowledge generation process is as follows. If *H*^*T*^*H* is nonsingular, the orthogonal projection method can be used to calculate the generalized inverse of a matrix (Huang et al., 2011b):


(5) $$\begin{array}{l} H^{+}\text{=}{\left(H^{T}H\right)}^{-1}H^{T} \end{array}$$

According to Equation 4 (Betthauser et al., 2017), we can get


(6) $$\begin{array}{l} \hat{\beta }\text{=}{\left(H^{T}H\right)}^{-1}H^{T}T \end{array}$$

For binary EEG classification applications, the decision function is:


(7) $$\begin{array}{l} f\text{(x) = sign(g(x)}\beta \text{)} \end{array}$$

g(.) maps the data from input space into the L-dimensional hidden-layer feature space (ELM feature space). By inserting Equations 6 into Equation 7, we can obtain


(8) $$\begin{array}{l} f\text{(x) = sign(g(x)}{\left(H^{T}H\right)}^{-1}H^{T}T\text{)} \end{array}$$

For multi-class EEG classification tasks, the corresponding predicted label of sample is the index number of the output node which has the highest output value for the given instance. *f*_*p*_ denotes the output function of *p*th node, then we have the predicted class label of sample *x*:


(9) $$\begin{array}{l} label\left(x\right)=arg\underset{p\in \left\{1,2,\ldots ,m\right\}}{max}f_{p}\left(x\right) \end{array}$$

Each sub-model in a higher layer takes information transformed from the decision output of the previous lower layers and appends them as supplementary knowledge, enabling more relevant representation to be handed over to the next generation. Deeper representation is captured to build a hierarchical network until the next additive ELM had no remarkable effect.

With deep representation in DELM, useful information is well-explored and transmitted from the initial layer to the last layer, bringing a more complete and precise expression of original input, improving the knowledge utilization rate greatly and strengthening the learning capability of ELM. Several ELMs are combined together by means of a serial link and the response can be reused in higher sub-model next to it. On the premise of meaningfulness of extended ELM, the purpose of the previous submodel is to convey the knowledge learned by previous layer. By updating the knowledge community, the original manifold can be separated apart in the end.

### 2.2. Knowledge augmentation based on DELM

**Figure 2:**
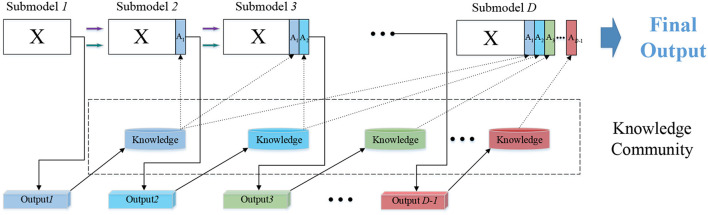
Stepwise knowledge learning in DELM.

A detailed introduction to knowledge transfer between multiple modules is provided in Figure 2. The input of *n* dimensional attributes provides data for the first level to construct a traditional ELM classifier. For *N* samples in a given dataset, *x*_*i*_ is the data of the *i*th dimension attribute corresponding to different samples, and *t*_*i*_ is the expected label, where $x_{i}={\left[x_{i1},x_{i2},\ldots ,x_{in}\right]}^{T}\in R^{n}$, $t_{i}={\left[t_{i1},t_{i2},\ldots ,t_{im}\right]}^{T}\in R^{m}$.

The expected label is expressed in *T* while the actual output *Y*_*d*_ calculated by the *d*th level model is expressed as:


(10) $$\begin{array}{l} Y_{d}=\left[\begin{array}{ccc} y_{11}^{d} & \ldots & y_{1N}^{d}\\ \vdots & \ddots & \vdots \\ y_{m1}^{d} & \cdots & y_{mN}^{d} \end{array}\right] \end{array}$$

*m* represents the number of categories of samples. The matrix form is as follows: $T={\left[t_{1},\cdots \;,t_{N}\right]}_{m\times N}^{T}$. After finishing the task of the first ELM, the output produced by sub-model1 is $Y_{1}\in R^{m\times N}$. Resemble the process in classic ELM, the output matrix should perform a transformation here. The information acquired by current sub-model is integrated, and the fused knowledge community is stored for the next knowledge transmission. For the *i*th instance, take the maximum value in its each column as its class label, store the class label $x_{n+1}\in R^{1\times N}$ and merge it with the original input. The updated input is obtained in the second level Submodel2: $x_{i}={\left[x_{i1},x_{i2},\ldots ,x_{in},x_{i\left(n+1\right)}\right]}^{T}\in R^{n+1}$. The label *T* remains the same as the original one. Similarly, calculate the actual output *Y*_2_. *Y*_2_ is transformed into knowledge again, and the significant information is stored in new input: $x_{i}={\left[x_{i1},x_{i2},\ldots ,x_{in},x_{i\left(n+1\right)},x_{i\left(n+2\right)}\right]}^{T}\in R^{n+2}$. Then, the third sub-model leverages knowledge extracted from the output of sub-model_1 and sub-model_2 to complete the classification of the model. Establish three modules or more on both training and testing sets and that can yield favorable results. The input for these modules comprises original features and appended features from all previous recognition prediction. So the augmented input for each module can be formed as:


(11) $$\begin{array}{l} X_{1}=X_{1}\\ X_{2}=\left[X_{1}|A_{1}\right]\\ X_{3}=\left[X_{2}|A_{2}\right]\\ \vdots \\ X_{D}=\left[X_{D-1}|A_{D-1}\right] \end{array}$$

At each level, the predicted output of current sub-model is integrated into the input as learning experiences. In the next learning step, the new input after incorporation will be mapped into a new ELM feature space through random mapping in current sub-model to solve the least square problem. The new features, including *A*_1_, *A*_2_ and so on, contains discriminative information derived from lower modules, so it is helpful in forcing the manifold structure apart in original EEG input. In this course of knowledge augmentation, DELM is aimed at learning a more reasonable decision basis from raw data in classification tasks.

### 2.3. Specialty of DELM pattern classifier

We are motivated by the idea of deep learning and stacking generalization theory, and establish a hierarchical ELM-based stacked architecture. Each sub-model has the same supervised learning process as classic ELM and several ELMs are integrated into a deep network. ELM in each level is an elegant original model, which is respectively composed of input layer, hidden layer and output layer in our paper. Under the guidance of the corresponding expected labels, DELM can better pull each sample to its own class cluster, hence, samples have a tendency to approach their own field gradually after knowledge augmentation. In other words, it makes it easier for the samples belonging to some class to be identified as belonging to its true class by DELM pattern classifier. Accordingly, the output generated in previous submodel performs knowledge transformation first, and then it is regarded as a supplement to the input. DELM is targeted at achieving a richer form of representation from raw data, which enables the sequential propagation of knowledge in a forward way and provides a method to automatically discover valuable implied patterns. With the valuable information extracted from the instances, the whole model is directed to study the internal information of instances, and constantly approach the ideal output with stepwise learning.

Noise caused by electrode movement or others often appears in the practical EEG signal, resulting in poor recognition results. The proposed framework has the anti-noise capability of deep network in practice in contrast to the traditional ELM algorithm, which can stand against the noise to a certain extent. With stepwise transformation of input EEG epileptic information, the dimension of the input expands continuously, and the pollution in the original data is gradually reduced or eliminated. Stepwise knowledge is continuously strengthened, more reasonable features are generated, and the final classification accuracy of epileptic EEG signals is improved.

The entire network consists of several stacked independent ELM modules. The stacked approach is one of the most effective ensemble learning strategies. Our model trains several submodels in a serial way, and each submodel still preserve the output of the previous submodel for deep representation learning, which shares the same philosophy as stacked generalization (Wolpert, 1992; Wang et al., 2017; Hang et al., 2020).

Our model is aimed at reducing the loss of effective information in the original data and greatly economizing the time required for classification under the premise of ensuring certain accuracy. The information is extracted, grows in refinement and richness, and is accepted to be vital members of the knowledge community ultimately. The sub-model that organizes the higher layer has additional input features involving the classification output from all previous sub-models. DELM learns reasonable and effective features from a large number of complex raw data, and the newly generated features are absorbed by our deep network into its own knowledge, which can achieve satisfactory results in most cases when faced with practical application problems.

In the previous phase, multi submodels are adopted for knowledge augmentation and knowledge are automatically captured through feature expansion. In the latter phase, the original input and the generated knowledge in previous modules are used to accomplish the modeling and the classification tasks. The deep learning algorithm of the proposed DELM is summarized in Algorithm 1.

**Algorithm 1:** DELM

**Input**: The dataset $S_{1}\text{=}\left\{\left(x_{i},t_{i}\right)|x_{i}\in R^{n},t_{i}\in R^{m},i=1,\ldots ,N\right\}$, where the original input matrix is expressed as *X*_1_, the activation function is g(.), total number of iterations is *r*.
**Output**: The output label *Y*.
**for** *k* = 1;*k* ≤ *r* **do**
Step 1:
(a) Randomly initialize input weights *w*_*i*_ and biases of hidden layer neurons *b*_*i*_;
(b) Calculate the output matrix of the hidden layer *H*_1_;
(c) Determine the output weights analytically according to Equation 6, ${\hat{\beta }}_{1}\text{=}H_{1}^{+}T$;
(d) Compute the classification results: $Y_{1}\text{=}H_{1}{\hat{\beta }}_{1}$, convert the actual output to label matrix *A*_1_, and store it into a new representation matrix *X*_2_ = [*X*_1_|*A*_1_], so the updated dataset of input: $S_{2}\text{=}\left\{{\left(x_{i},t_{i}\right)|x_{i}\in R}^{n+1},t_{i}\in R^{m},i=1,2,\ldots ,N\right\}$.
Step 2: Initialize the depth *d* = 2. **Repeat**
(a) Randomly initialize input weights and biases of hidden layer neurons;
(b) Calculate the new output matrix of the hidden layer *H*_*d*_, *d* refers to the *d* th submodel of the current training process;
(c) The output weight of corresponding submodel is calculated: ${\hat{\beta }}_{d}\text{=}H_{d}^{\text{+}}T$;
(d) Compute the classification output: $Y_{d}\text{=}H_{d}{\hat{\beta }}_{d}$, the matrix after label transformation of output *A*_*d*_, and store it into a new representation matrix *X*_*d*+1_ = [*X*_*d*_|*A*_*d*_], so the updated dataset of input: $S_{d+1}\text{=}\left\{{\left(x_{i},t_{i}\right)|x_{i}\in R}^{n+d},t_{i}\in R^{m},i=1,2,\ldots ,N\right\}$. (e) Set *d* = *d*+1.
**until** the testing error threshold between the two adjacent submodels is satisfied

### 2.4. Time complexity analysis

In order to exhibit the time complexity of the proposed deep learning algorithm, we start with the classic ELM algorithm first. The time complexity of classic ELM algorithm mainly lies in the solution of Moore-Penrose generalized inverse of hidden output matrix. In terms of Equation 5, *O*(*N*^2^*L*) can be required to compute the *H*^*T*^*H*. It requires *O*(*N*^3^) to calculate the inverse. So the time complexity in ELM becomes *O*(*N*^3^ + *N*^2^*L* + *NnL* + 1). The proposed DELM introduces the concept of deep learning, which is composed of several building units. Obviously, the time complexity of the entire DELM can be indicated as $\text{O}\left(\sum_{d=1}^{D}\left(N^{3}+N^{2}L+NnL+1\right)\right)$, where *D* is the final value of depth, *L* is the number of hidden layer neural network units and *N* is the number of instances.

## 3. Experiment studies

In this section, we will demonstrate the effectiveness of our proposed hierarchical model DELM by reporting the experiment result from Bonn dataset. In our experimental study, DELM is sequentially compared with some machine learning algorithms and popular deep learning networks such as DBN, and so on. The final performance evaluation is performed according to the result. In our experiment, all adopted methods were implemented using MATLAB 2019a on a personal computer with Intel Core i5-9400 2.90 GHz CPU and 8.0G RAM.

### 3.1. Epileptic EEG dataset

The EEG signals used in the paper are derived from Department of Epileptology, Bonn University, Germany. The dataset has been described in detail by Andrzejak et al. (2001). The EEG signals were collected under various conditions with five healthy volunteers and five epileptic patients. The details information of five groups are summarized in Table 2, in which each group contains 2,300 samples.

**Table 2:** A brief introduction to the EEG dataset.

**Condition**	**Set**	**Description**
Healthy volunteer	A	EEG signals obtained from healthy volunteers with their eyes open.
	B	EEG signals obtained from healthy volunteers with their eyes closed.
Epileptic volunteer	C	EEG signals obtained from the hippocampal formation of the opposite hemisphere of the brain during seizure free intervals.
	D	EEG signals obtained from the epileptogenic zone during seizure free intervals.
	E	EEG signals obtained during the onset of epileptic seizure.

**Figure 3:**
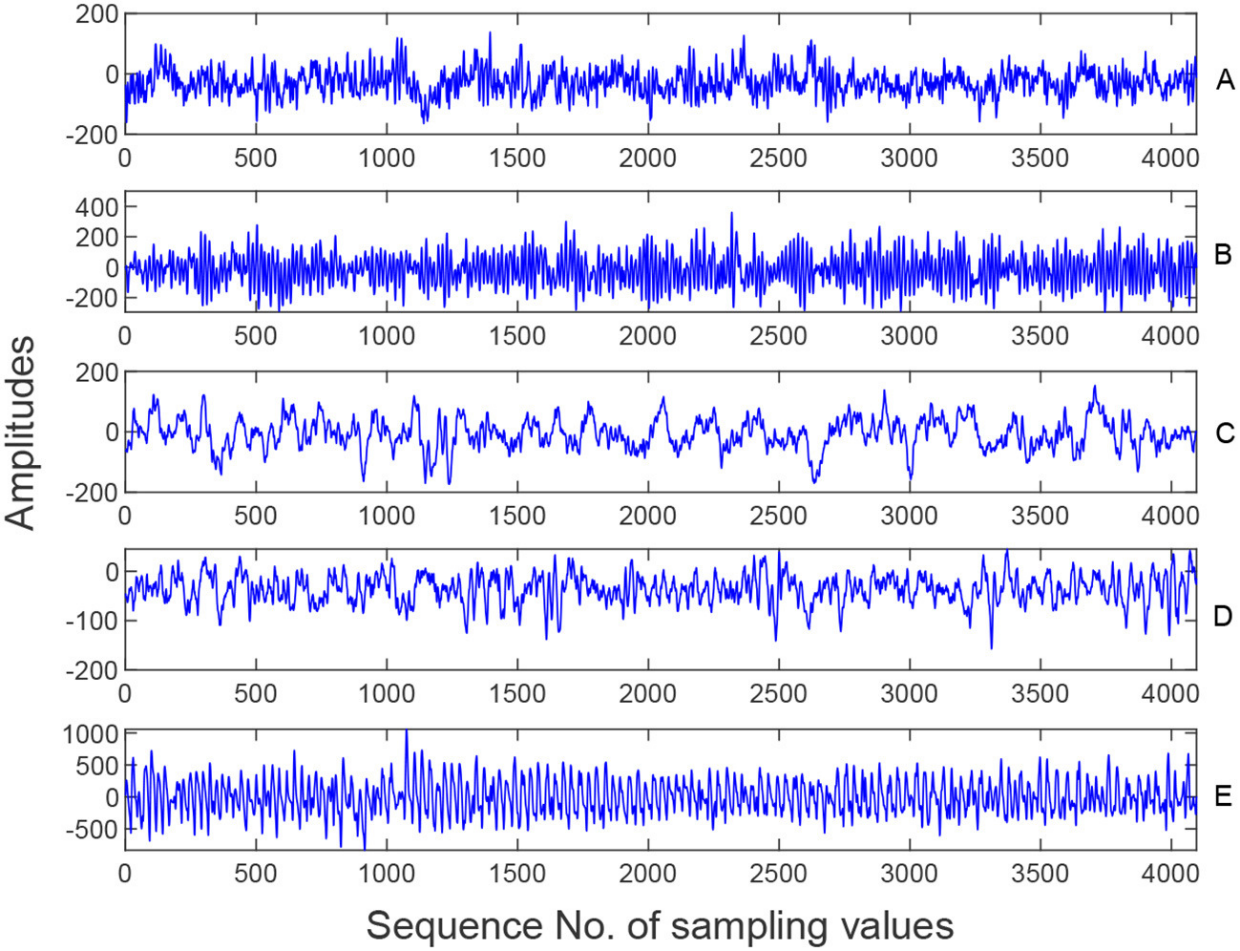
Samples from five EEG sets **(A–E)**.

The dataset consists of five groups of data (A, B, C, D, and E) where each containing 100 single-channel EEG segments. EEG data were recorded using the same 128-channel amplifier system with a sampling rate of 173.6 Hz and a 12-bit resolution. Each EEG segment contained 4,096 sampling points and lasted 23.6 s. The five samples in Figure 3 come from Set A, B, C, D and E respectively, as shown below.

In our experiment, three kinds of EEG signals are employed, namely normal (A and B), interictal (C and D), and ictal (E), to evaluate the proposed epilepsy detection framework.

### 3.2. Data preparation and normalization

Firstly, the EEG signals are segmented into 178 sampling points by means of moving windows, among which there is no overlapping of sampling windows. Therefore, 23 epochs can be obtained from each segment. The remaining points in each segment are dismissed. Different features extracted from the original EEG signals have different scales after data segmentation, so it is necessary to use normalization processing to normalize all attribute features.

### 3.3. Experiment setup

In our experimental organization, the processed dataset is firstly randomly divided into two parts: training and testing set. In each scenario, we randomly selected 80% of the data as the training data, and the remaining 20% as the testing data. The experiment is repeated 20 times in various scenarios and then the average experimental results of some other schemes are also collected as contrast. In our experiment, SVM, RBF and some ensemble algorithms such as Adaboost are used. Meanwhile, experimental results of well-known deep networks, such as DBN and SAE, are also adopted as comparison in our experiment in order to demonstrate the superiority of the proposed DELM.

To reasonably evaluate our method, the performance metrics adopted here are *Accuracy* and *F*−*measure*, which are defined as follows:


(12) $$\begin{array}{l} Accuracy\text{=}\frac{TP+TN}{TP+TN+FP+FN}; \end{array}$$

(13) $$\begin{array}{l} F-measure\text{=}\frac{2\times TP}{2\times TP\text{+}FN\text{+}FP}; \end{array}$$

where TP (true positive) represents the number of segments detected as seizure correctly, FN (false negative) represents the number of segments detected as non-seizure incorrectly, TN (true negative) represents the number of segments detected as non-seizure correctly, and FP (false positive) is the number of segments detected as seizure incorrectly.

In terms of recognition accuracy, our DELM model can achieve great classification accuracy comparable to that of deep learning schemes. Running time is one of the key evaluation indexes which can perform excellent performance in DELM. The classic ELM is qualified for real-time recognition requirements and so does our hierarchical model. Extremely fast recognition ability can still be exhibited in DELM, meanwhile traditional deep networks are too far behind to catch with.

Among all the competing schemes, SVM, RBF, and ensemble algorithms were implemented by toolbox in MATLAB. And traditional deep learning algorithms are implemented by MATLAB which is encapsulated in the DeepLearning Toolbox. The parameters settings are summarized in Table 3.

**Table 3:** The detailed parameters used in our experiment.

**Algorithm**	**Parameter description**
SVM	*c*∈{2^−4^, 2^−3^, 2^−2^, 2^−1^, 1, 2, 2^2^, 2^3^}
	*g*∈{2^−5^, 2^−4^, 2^−3^, 2^−2^, 2^−1^, 1, 2}
RBF	*spread*∈{2^−1^, 2^0^, 2, 2^2^, 2^3^, 2^4^}
Adaboost	*NLearners*∈{5, 10, 15, 20, …, 100, 200, 1000}
Bagging	*NLearners*∈{2, 3, 4, 5}
DBN	*numepochs*∈{30, 40}
	*batchsize*∈{20, 40, 80}
SAE	*numepochs*∈{20, 30, 40, 50}
	*batchsize*∈{20, 40, 80}

In each sub-model, all input weights and hidden biases are set to the pseudo random values drawn from the uniform distribution on the interval (−1, 1) and (0, 1). Such scheme is in accordance with the standard methodology of ELM, which simplifies the learning process. In each ELM, the hidden layer adopts the same number of hidden nodes and the same activation function. Sigmoid function is chosen as the activation function g(.) in each submodel. The number of hidden units is usually scenario-specific and determined by experience or by continuous attempts. We need to find a point as balanced as possible between the number of hidden units and time. As a result, DELM can acquire a relatively mature knowledge system, which can well meet the accuracy requirements of classification. The optimal amount of hidden units in all sub-models is uniformly set to a fixed value 500. Considering the difficulty of recognition in five class problem, the number of hidden units is set to 800. More ELMs can be cascaded to modules, if desired, for the purpose of adequate knowledge. So we dynamically determine the depth of network. The stacking process will be aborted if the difference between the current and upper level in the experiment is <0.1. It is clear that DELM simply involves a few parameters, which greatly reduces the cost of parameter adjustment. To evaluate DELM comprehensively and precisely, classification tasks in various scenarios are designed here.

### 3.4. Epileptic EEG signal recognition

#### 3.4.1. Two class problem

Classification of four combinations between A and E, B and E, C and E, and D and E are considered to distinguish normal from seizure. Epileptic seizure segments E was selected to compare with one of the remaining EEG sets from the dataset for classification. Then select two or more sets in the database and conduct trials again. The combinations are as follows: AB and E, AC and E, AD and E, BC and E, BD and E, CD and E, ABC and E, ABD and E, ACD and E, BCD and E, and ABCD and E.

#### 3.4.2. Three class problem

In three class problems, the selected combinations are: A, B, E and A, C, E and A, D, E and B, C, E and B, D, E and C, D, E.

#### 3.4.3. Five class problem

In five class problem, each group is regarded as an independent class for testing.

### 3.5. Experimental results and statistical analysis

Table 4 shows us the accuracy in the sense of both the mean and standard deviation in DELM and deep networks. The results are also presented when the depth *d* of DELM is 3. But the result in the case is still a certain gap from the ideal, and more ELMs are required to assure higher accuracy. In terms of accuracy, DELM can compete with conventional intelligent methods. It can be noted in the results that the proposed method has certain advantages over traditional methods and is generally comparable to traditional deep networks. We attribute this advancement in recognition performance to the embedded knowledge. The accuracy is greatly improved by extending the vertical network layers and the model gradually acquires a better command of the implication of knowledge. Table 4 also report the accuracy of common machine learning algorithms on our datasets.

**Table 4:** Average testing accuracy in our experiment.

	**SVM**	**RBF**	**Adaboost**	**Bagging**	**DBN**	**SAE**	**Basic ELM**	**DELM (D = 3)**	**DELM**
A/E	0.9277	0.8745	0.9317	**0.9443**	0.9279	0.9123	0.8866	0.9174	0.9307
	(0.0080)	(0.0064)	(0.0147)	(0.0099)	(0.1501)	(0.0095)	(0.0100)	(0.0051)	(0.0067)
B/E	0.9067	0.8678	0.8727	0.9020	0.9086	0.9039	0.8754	0.9091	**0.9180**
	(0.0065)	(0.0087)	(0.0082)	(0.0152)	(0.0083)	(0.0181)	(0.0154)	(0.0073)	(0.0091)
C/E	0.9135	0.8641	0.9007	0.9165	0.8978	0.9098	0.8764	0.9050	**0.9196**
	(0.0089)	(0.0087)	(0.0061)	(0.0076)	(0.0350)	(0.0125)	(0.0102)	(0.0124)	(0.0065)
D/E	0.8603	0.8311	0.8730	0.8887	0.8993	0.8545	0.8400	0.8737	**0.9137**
	(0.0083)	(0.0067)	(0.0115)	(0.0076)	(0.0219)	(0.0134)	(0.0093)	(0.0103)	(0.0096)
AB/E	0.9377	0.9028	0.9116	0.9305	0.9286	0.9345	0.9055	0.9326	**0.9415**
	(0.0057)	(0.0020)	(0.0059)	(0.0117)	(0.0271)	(0.0101)	(0.0065)	(0.0058)	(0.0070)
AC/E	0.9004	0.9023	0.9333	0.9341	0.9032	0.9388	0.9043	0.9301	**0.9395**
	(0.0091)	(0.0067)	(0.0076)	(0.0056)	(0.0778)	(0.0044)	(0.0057)	(0.0041)	(0.0050)
AD/E	0.8746	0.8832	0.9106	0.9249	**0.9354**	0.9042	0.8796	0.9078	0.9157
	(0.0086)	(0.0092)	(0.0099)	(0.0091)	(0.0126)	(0.0062)	(0.0077)	(0.0065)	(0.0073)
BC/E	0.8978	0.8972	0.9084	0.9201	0.9357	0.9102	0.9003	0.9269	**0.9367**
	(0.0105)	(0.0075)	(0.0081)	(0.0060)	(0.0099)	(0.0086)	(0.0094)	(0.0072)	(0.0074)
BD/E	**0.9220**	0.8830	0.8834	0.9001	0.9210	0.9002	0.8767	0.9050	0.9111
	(0.0056)	(0.0068)	(0.0078)	(0.0067)	(0.0089)	(0.0089)	(0.0099)	(0.0079)	(0.0076)
CD/E	**0.9312**	0.8788	0.8993	0.9151	0.9280	0.9033	0.8802	0.9085	0.9122
	(0.0052)	(0.0079)	(0.0053)	(0.0035)	(0.0089)	(0.0081)	(0.0098)	(0.0083)	(0.0056)
ABC/E	0.9390	0.9254	0.9263	0.9398	0.9347	0.9370	0.9260	0.9460	**0.9497**
	(0.0046)	(0.0052)	(0.0066)	(0.0046)	(0.0580)	(0.0055)	(0.0051)	(0.0048)	(0.0055)
ABD/E	0.9202	0.9076	0.9154	0.9284	**0.9556**	0.9334	0.9067	0.9277	0.9320
	(0.0063)	(0.0030)	(0.0064)	(0.0050)	(0.0058)	(0.0072)	(0.0073)	(0.0060)	(0.0058)
ACD/E	0.9243	0.9102	0.9228	0.9324	0.9269	0.9285	0.9098	0.9282	**0.9329**
	(0.0061)	(0.0034)	(0.0064)	(0.0052)	(0.0627)	(0.0060)	(0.0055)	(0.0071)	(0.0051)
BCD/E	0.9209	0.9085	0.9112	0.9176	**0.9536**	0.9424	0.9061	0.9277	0.9299
	(0.0057)	(0.0088)	(0.0052)	(0.0076)	(0.0038)	(0.0032)	(0.0058)	(0.0048)	(0.0056)
ABCD/E	0.9390	0.9212	0.9243	0.9357	0.9224	0.9418	0.9235	0.9396	**0.9442**
	(0.0028)	(0.0044)	(0.0089)	(0.0060)	(0.0066)	(0.0069)	(0.0073)	(0.0046)	(0.0035)
A/B/E	0.7406	**0.7519**	0.6392	0.6894	0.7225	0.6746	0.6919	0.7071	0.7142
	(0.0134)	(0.0158)	(0.0223)	(0.0111)	(0.0422)	(0.0172)	(0.0086)	(0.0120)	(0.0107)
A/C/E	**0.6975**	0.6665	0.6481	0.6771	0.6829	0.6717	0.6524	0.6750	0.6797
	(0.0123)	(0.0169)	(0.0262)	(0.0132)	(0.0387)	(0.0194)	(0.0084)	(0.0104)	(0.0128)
A/D/E	0.6530	0.6322	0.6457	0.6738	0.6329	0.6673	0.6436	0.6658	**0.6708**
	(0.0107)	(0.0133)	(0.0259)	(0.0114)	(0.0319)	(0.0191)	(0.0104)	(0.0099)	(0.0116)
B/C/E	0.7052	0.6275	0.6058	0.7193	0.6839	0.6812	0.7176	0.7350	**0.7371**
	(0.0124)	(0.0148)	(0.0192)	(0.0115)	(0.0421)	(0.0215)	(0.0141)	(0.0103)	(0.0166)
B/D/E	0.6641	0.5032	0.5838	0.7143	**0.7288**	0.6486	0.6717	0.6961	0.7002
	(0.0124)	(0.0210)	(0.0073)	(0.0134)	(0.0231)	(0.0168)	(0.0107)	(0.0142)	(0.0107)
C/D/E	0.6278	0.5787	0.6060	0.6262	0.6263	0.6045	0.6068	0.6273	**0.6306**
	(0.0152)	(0.0077)	(0.0086)	(0.0102)	(0.0075)	(0.0100)	(0.0147)	(0.0097)	(0.0134)
A/B/C/D/E	0.4954	0.3557	0.3994	0.4058	0.4883	0.4227	0.4585	0.4722	**0.5058**
	(0.0048)	(0.0098)	(0.0116)	(0.0126)	(0.0239)	(0.0184)	(0.0087)	(0.0102)	(0.0127)

The best results are marked in bold.

Since DELM can inherit advantages of ELM, extremely fast learning speed is one of its remarkable characteristics. In the aspect of computational efficiency, the slight increase of learning time (extremely short seconds) in DELM compared to the original ELM is inappreciable, especially when considering the added improvement in classification accuracy. DELM is about sacrificing a little time and tolerating a cascade of multi modules in exchange for final performance, so we just need to draw comparison between our ELM-based deep network and traditional deep network. The experimental results show that the time needed in DELM is much less than that of the traditional deep networks after the accuracy is guaranteed to meet the requirements. In some designed scenarios, the speed of DELM in training and testing is approximately a dozen times faster than traditional deep networks.

**Figure 4:**
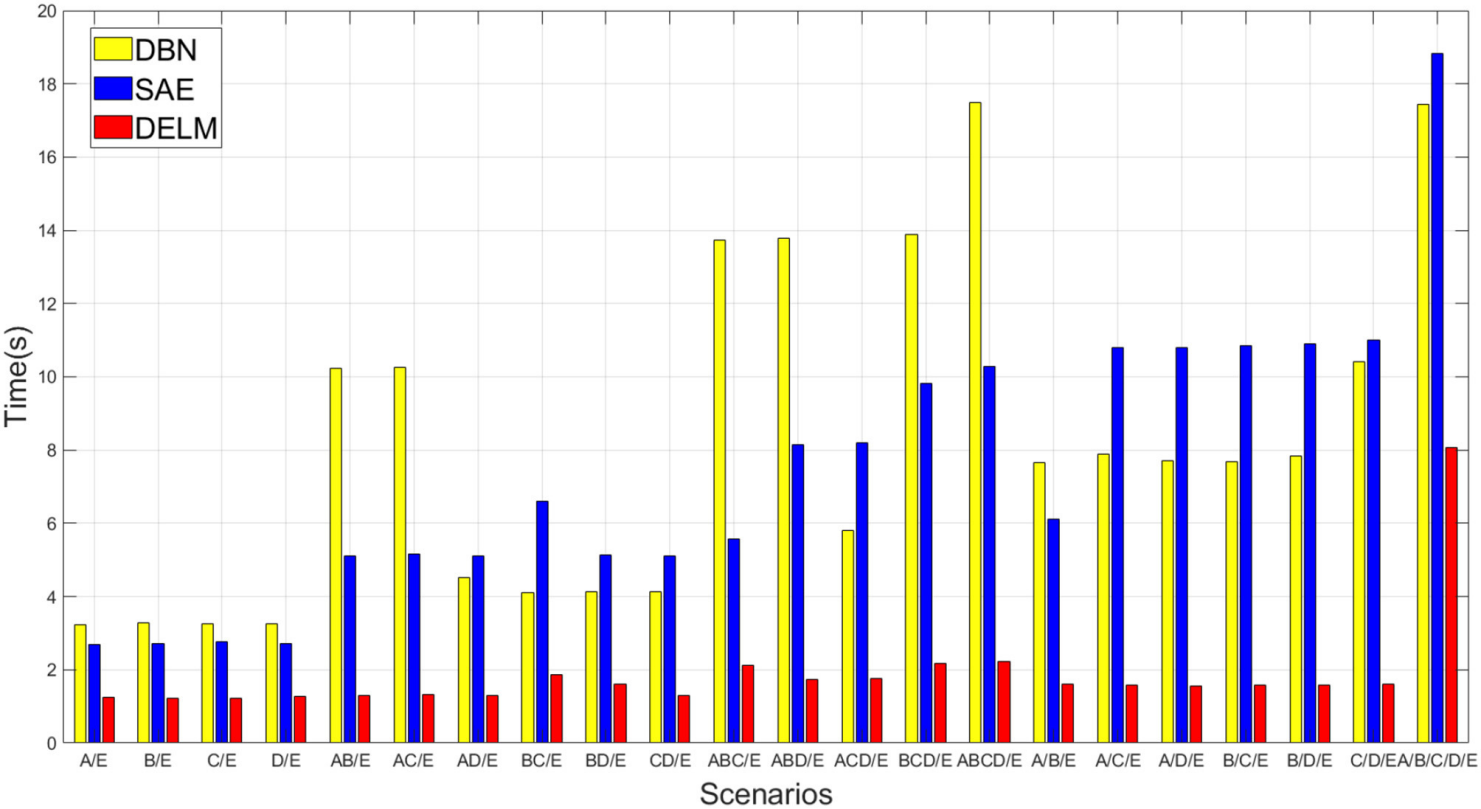
Average time consumed by deep networks in our experiment.

Figure 4 reports time efficiency during learning process, and the result is average learning time of models. As observed from both Table 4 and Figure 4, the accuracy performance is almost similar in DELM and traditional deep methods. However, the time consumed by the proposed classifier is the least. Taking into account both accuracy and computational effort simultaneously, the proposed DELM demonstrates tremendous potential in EEG classification and may be a competitive choice.

**Figure 5:**
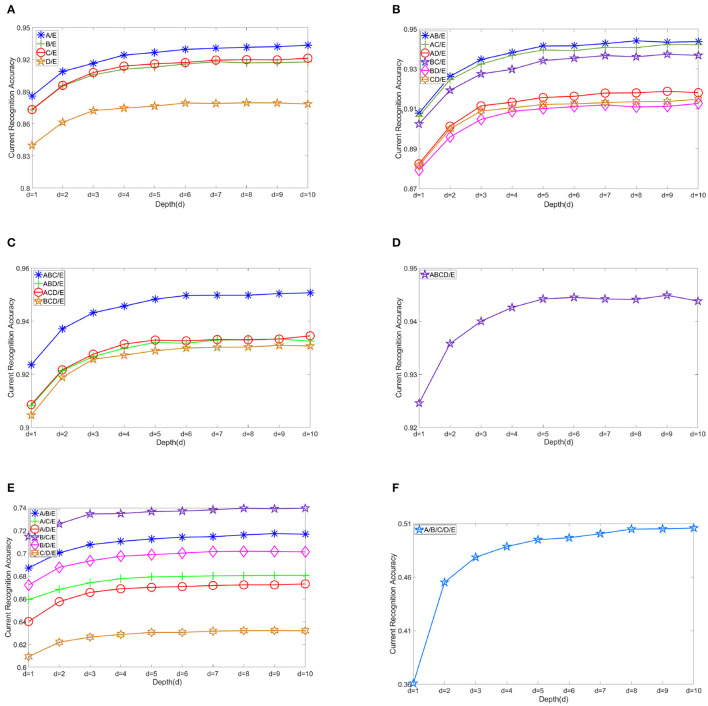
Changes in recognition accuracy along with stacked depth of modules in various scenarios. **(A)** Another individual group vs. ictal A/E, B/E, C/E, and D/E. **(B)** Two other groups vs. ictal AB/E, AC/E, AD/E, BC/E, BD/E, and CD/E. **(C)** Three other groups vs. ictal ABC/E, ABD/E, ACD/E, and BCD/E. **(D)** All other groups vs. ictal ABCD/E. **(E)** A/B/E, A/C/E, A/D/E, B/C/E, B/D/E, and C/D/E. **(F)** A/B/C/D/E.

Figure 5 shows the changes in recognition accuracy along with current stacked depth of modules in different EEG classification scenarios. There is no doubt that the EEG classification accuracy increases with the addition of sub-models. The number of submodels we use is namely the depth of DELM. Depth is denoted by *d*, and the result shows the classification accuracy from *d* = 1 to *d* = 10. It is shown that the improvement in accuracy can be relatively evident in the first three levels. Modest improvement can still be obtained in the subsequent expansion of ELMs, but DELM will gradually lose competitiveness in real-time tasks. Without rapid classification performance, what we think of as our inherent excellence in our model, several serial ELM network modules in our model, would make no sense and our previous efforts would not worth it. By starting from *d* = 1, ordinary extreme learning machine, excellent features can be well-preserved and classification effect is gradually improved. Performance augmentation can be seen in these figures.

**Figure 6:**
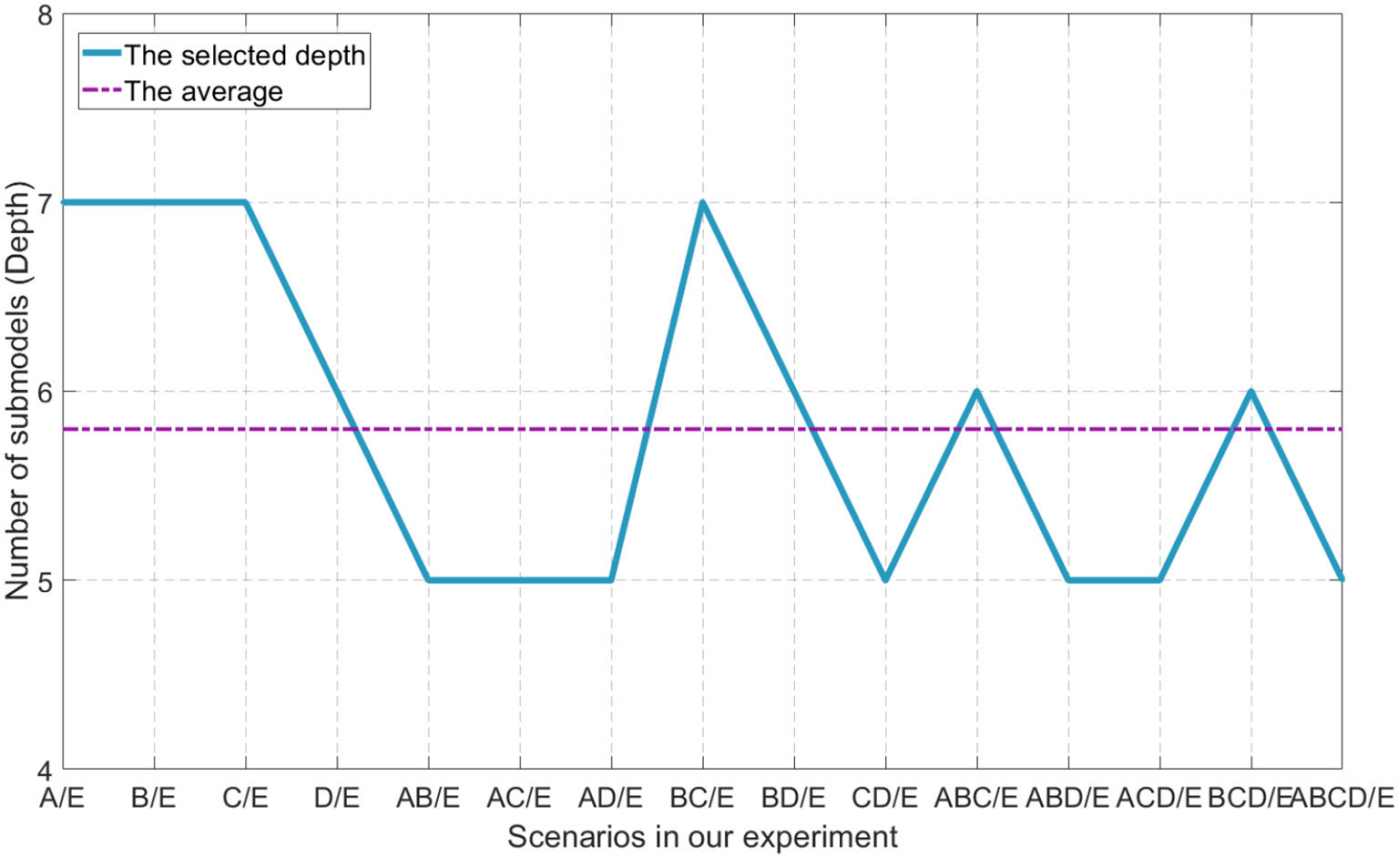
Different parameters of depth used in our experiment.

The depth is the key aspect to knowledge augmentation in DELM. In our experimental organization, different depths are adopted in binary class problems, while *D* = 6 is uniformly adopted in three and five class problems in order to obtain better classification accuracy. Setting a threshold for DELM is because excessive accumulation of layers is not productive any more. The average depth of binary class problems is *d*_*AVG*_ = 5.8. The selection of depth parameters are shown in Figure 6.

Table 5 presents *F*−*measure* scores obtained by traditional deep learning methods in different scenarios. From the perspective of *F*−*measure* scores, DELM outperforms several deep networks used for comparison. In other scenarios, DELM is slightly worse than deep networks, but it still performs well and is comparable to deep networks.

**Table 5:** *F*−*measure* scores of the comaparative methods.

**Method**	**SVM**	**RBFN**	**Adaboost**	**Bagging**	**DBN**	**SAE**	**DELM**
A/E	0.9225	0.8560	0.9287	**0.9431**	0.8784	0.9050	0.9262
B/E	0.8954	0.8449	0.8637	0.9071	0.9102	0.8943	**0.9103**
C/E	0.9048	0.8450	0.8961	**0.9215**	0.9071	0.9016	0.9122
D/E	0.8481	0.8120	0.8690	0.8953	0.8985	0.8388	**0.9056**
AB/E	0.8975	0.8290	0.8544	0.9024	0.8811	0.8916	**0.9027**
AC/E	0.8200	0.8271	0.8923	0.8998	0.8091	0.8997	**0.9000**
AD/E	0.7727	0.8001	0.8550	0.8961	**0.9037**	0.8407	0.8597
BC/E	0.8177	0.8240	0.8480	0.8886	0.8916	0.8443	**0.8939**
BD/E	0.8716	0.7986	0.8060	0.8612	**0.8799**	0.8356	0.8525
CD/E	**0.8892**	0.7880	0.8334	0.8830	0.8886	0.8366	0.8536
ABC/E	0.8594	0.8216	0.8384	0.8875	0.8287	0.8584	**0.8888**
ABD/E	0.8172	0.7830	0.8142	0.8667	**0.9112**	0.8524	0.8473
ACD/E	0.8274	0.7897	0.8333	**0.8769**	0.8048	0.8415	0.8511
BCD/E	0.8203	0.7872	0.8016	0.8486	**0.9054**	0.8785	0.8445
ABCD/E	0.8251	0.7695	0.7884	0.8524	**0.9120**	0.8412	0.8447
A/B/E	0.7268	**0.7573**	0.5920	0.6809	0.6853	0.6677	0.7151
A/C/E	0.6774	0.6752	0.5975	0.6602	0.6366	0.6429	**0.6836**
A/D/E	0.5872	0.6414	0.6083	**0.6578**	0.6266	0.6229	0.6476
B/C/E	0.6924	0.6237	0.5518	0.7037	0.6421	0.6705	**0.7405**
B/D/E	0.6677	0.5121	0.5375	0.7002	**0.7124**	0.6257	0.7074
C/D/E	0.5800	0.5874	0.5990	0.6034	0.5484	0.5873	**0.6274**
A/B/C/D/E	0.4709	0.3163	0.3411	0.4013	0.4494	0.3849	**0.5047**

The best results are marked in bold.

DELM enjoys extremely fast speed of ELM while providing deeper representation of original signals. Experiments show that our algorithm consistently outperforms several existing state-of-art schemes in terms of accuracy and execution time.

## 4. Conclusion

A novel deep extreme learning machine DELM is proposed for the recognition of EEG epileptic signals in our paper. DELM stepwisely transmits the response to the next submodel through fusion of knowledge derived from previous sub-models. Such a process is beneficial to mine the valuable information of the original EEG data, so as to better accomplish the subsequent EEG recognition tasks. The proposed model operates in a forward way with an increment form to strive for an increasingly efficient performance and its computation speed is considerably fast. ELM is introduced as the basic building block, making the whole learning process flexible and effective. As available knowledge, the classification results of the previous multi-module can enhance the classification performance of the subsequent modules. Our experimental results demonstrate that the proposed method is a promising candidate for epileptic EEG-based recognition. Compared with traditional methods, the proposed DELM is motivated by deep learning and stack generalization theory, which can obtain excellent classification results and outperform the traditional methods. According to stacking generalization theory, the output of the next sub-model plus the knowledge of the previous sub-model in DELM can indeed open the manifold structure of the input space, which resulting an improved performance. Moreover, knowledge augmentation can effectively extract the implied knowledge in each sub-model and obtain increasing performance.

However, it is still not clear the reason for the improvement of knowledge augmentation throughout the training process. In the future work, we will spare no efforts to theoretically demonstrate how the prediction output in each ELM module can be helpful with EEG epileptic signal recognition.

## Data availability statement

The datasets presented in this study can be found in online repositories. The names of the repository/repositories and accession number(s) can be found at: http://epileptologie-bonn.de/cms/upload/workgroup/lehnertz/eegdata.html.

## Author contributions

XZ and JZ conceived and designed the theoretical framework of this paper. All authors participated the experimentation and analysis process and drafted the manuscript. All authors contributed to the article and approved the submitted version.
